# Variational Autoencoder for the Prediction of Oil Contamination Temporal Evolution in Water Environments

**DOI:** 10.3390/s25061654

**Published:** 2025-03-07

**Authors:** Alejandro Casado-Pérez, Samuel Yanes, Sergio L. Toral, Manuel Perales-Esteve, Daniel Gutiérrez-Reina

**Affiliations:** Department of Electronics Engineering, University of Seville, 41009 Seville, Spain; syanes@us.es (S.Y.); storal@us.es (S.L.T.); mperales@us.es (M.P.-E.); dgutierrezreina@us.es (D.G.-R.)

**Keywords:** VAE, prediction, neural networks, contamination model

## Abstract

The water quality monitoring of large water masses using robotic vehicles is a complex task highly developed in recent years. The main approaches utilize adaptative informative path planning of fleets of autonomous surface vehicles and computer learning methods. However, water monitoring is characterized by a highly dynamic and unknown environment. Thus, the characterization of the water quality state of a water mass becomes a challenge. This paper proposes a variational autoencoder structure, trained in a model-free manner, that aims to provide a dynamic contamination model from partial observations of a homogeneous fleet of autonomous surface vehicles. To train the proposed approach, an oil spillage simulator based on heuristics is provided for world building. The proposed variational autoencoder was tested in three different environments characterized by different oil spill movements and twp different fleets equipped with different sensors. The results show accurate future contamination distribution predictions with a mean squared error ranging from 3 to 9% of the baseline at validation, defined as the static approach. Further tests addressed the overfit of the proposed neural network, showing a high robustness against unseen scenarios, and the effects of the gathered monitoring information in the variational autoencoder performance.

## 1. Introduction

Water plays an irreplaceable role in activities like the conservation of biodiversity, agriculture, tourism and industry, among others. However, besides being the most abundant liquid on the planet, there is a severe scarcity of quality usable water. This problem is becoming more accentuated due to climate change in recent years [1], requiring huge investments and difficult treatments for finding new water sources and acquiring quality water. The United Nations reflected the need for cooperation between worldwide organizations in Sustainable Development Goal (SDG) 6 [2]. This goal aims to ensure the availability and sustainable management of water and sanitation for all. SDG target 6.3 addresses another problem related to the release of hazardous chemicals and materials.

Approximately half of all the wastewater generated worldwide is released without treatment [3]. This, in addition to the accidental waste of residues like organic matter, oil spillage, heavy metals, and even radioactive substances, makes the situation a real environmental hazard. If these sources of contamination are not detected and treated accordingly in time, they can extend and cover the whole water surface, contaminating it directly, affecting its biodiversity, or indirectly promoting the appearance of invasive species or algae blooms [4], which, with time, can make the water unhealthy for human use. To avoid reaching this state of environmental crisis, water quality values must remain within water quality standards [5], and governments and organizations need to continuously monitor water masses. Monitoring is the preemptive measure against water contamination and degradation, as recovery is a process that takes several years [6].

Traditional water quality monitoring approaches focus on taking manual measures and analyzing samples in laboratories, requiring a lot of effort and human resources [7]. Recently, traditional methods are being replaced with superficial satellite methods [8] or intelligent robots, such as submarines and surface or aerial vehicles, that can be equipped with water quality sensors and robotic actuators [7]. Thus, vehicles are able to perform tasks ranging from exploration to actuation on water masses [9], involving detection, chasing and cleaning pollutants, and other monitoring tasks in real time. Furthermore, the time taken in laboratories to analyze samples induces a delay that, in the case of emergent contaminants, can cause a public health problem [10] not present when monitoring with autonomous vehicles. Therefore, it is envisioned that autonomous vehicles will play a crucial role in SDG target 6.6 in protecting water masses bodies [2].

The improvements in battery autonomy and computation power have made autonomous vehicles able to take intelligent actions. Thus, tasks that previously required an operator remotely controlling the vehicle are being replaced with a programmed movement policy that dictates the behavior of the vehicle [11]. The objective of these policies is to provide vehicles with target points or waypoints to travel to, making obtaining a policy a path planning problem. Another objective is to optimize the monitoring task assigned, which can be exploration or actuation, while taking into account factors present in the vehicle, such as battery, sensing, and actuating constraints. Thus, the path planning problem needs to take into account information about the environment. Developing a policy becomes a complex challenge due to the highly dynamic scenario of water masses. Since water is a fluid affected by several forces that facilitate the movement of particles through the whole mass, determining how a mass of water and its properties evolve through time is difficult. Therefore, vehicles need to adapt to this environment. Vehicles do not have prior knowledge about the environment. As a consequence, information is gathered by the vehicles during its mission and processed inside the vehicle or at a base station that the vehicle is able to communicate with, making offline planning an invalid solution.

With the advancements of neural networks, this field was able to provide solutions to the Adaptative Informative Path Planning (AIPP) problem based on deep architectures that have been developed [12]. As a clear example, Deep Reinforced Learning (DRL) approaches are able to solve the informative path planning problem, providing a valid policy with which vehicles are able to carry out the designated task [12], offering more robust and scalable solutions that adapt to the complexities and uncertainties of the environment. There are various optimization tasks, but regarding AIPP algorithms, some previous works have focused on water quality monitoring, contamination phenomenon exploration, and search tasks [13]. Among these previous works, some have focused on the contamination detection of algae blooms [14] and oil spills [15] using autonomous surface vehicles (ASVs), which are also called agents in the field of AIPP [16], equipped with specialized sensors [17,18]. However, most of the previous works made the assumption of lentic waters [19]. This means that the evolution of water properties and contaminants is slow and it can be considered that they do not change throughout a monitoring mission. However, this is not the case in larger water bodies like seam rivers and larger lakes, where the scenario is highly dynamic due to currents and wind, among other factors. Therefore, water quality conditions may evolve at the same time or faster than the actuation of the vehicles, and consequently, measures can become easily outdated. Although the use of multiple vehicles can alleviate the problem by increasing the data samples [20] or processing the age of the data collected [21], the obtained models will not reflect the real evolution of water quality parameters. Therefore, the planning actions will be sub-optimal in real scenarios.

The aim of a contamination model is to solve the estimation problem, obtaining the whole contamination map from partial observations and estimating its evolution. Several contamination models have been studied in the past [22]. In [23], the evolution of a contaminant in a river was modeled using mathematical hydrodynamic equations and solving the inverse model, reducing the potential harm caused by pollution accidents. In [24], several numerical models based on advection–dispersion equations or transport models for vulnerability assessment were used. However, characterizing the evolution in a larger water mass like a lake with partial observations cannot be explicitly described with equations, as it is affected by several chaotic effects. Bayesian contamination models like the Gaussian process [25] are able to provide valid solutions to the static problem, with the downside of a high computational cost that increases with the number of samples. In [26], a contamination model was obtained using a variational autoencoder (VAE) neural network, providing a more scalable solution with water samples. In the same paper, the results showed that a good contamination model is able to provide improvement in policy performance of approximately 50%. Thus, offering a forecasting module of the contamination that provides not only the present state of the contamination but makes a prediction of the future state of contaminants is likely to improve policy performance even further.

This paper proposes a variational autoencoder architecture based on the popular UNet network [27] combined with a prior and posterior convolutional neural network (CNN) architecture [28]. In [26], a similar architecture was proposed for the static case. In this paper, it was extended to a dynamic case, analyzing its capacity to estimate future distributions. This network was trained in a model-free manner, using only simulated interactions of the agents with the environment. The aim of the simulator is to provide a spatio-temporal distribution of pollutants in water bodies, replicating an oil spill accident. The simulator was used to create training and test datasets. The proposed VAE-UNet architecture will be a tool for any AIPP algorithm to plan ahead. The VAE works as a model that can provide accurate information about the contamination state from partial observations. Simultaneously, it captures the temporal-dependent behavior of the contamination, providing foresight for future contamination states.

To summarize, this paper contributes the following:

- A novel VAE neural network following the U-Net architecture that aims to provide future state estimations of water pollutant evolution.
- A comparison of the network performance against a naive baseline prediction.
- A further study of the limitations and overfitting of the suggested architecture.

This paper is organized as follows: Section 2 presents the materials and methods and describes the problem that the proposed VAE wants to solve, as well as how to set up the environment, contamination, agents, and the simulator. Lastly, the architecture of the VAE will be discussed. In Section 3, the results of the VAE obtained will be analyzed, and the model’s behavior will be compared with that of a naive model. In Section 4, the main contributions of this paper and future lines of work will be discussed.

## 2. Materials and Methods

### 2.1. Problem Formulation

**Figure 1:**
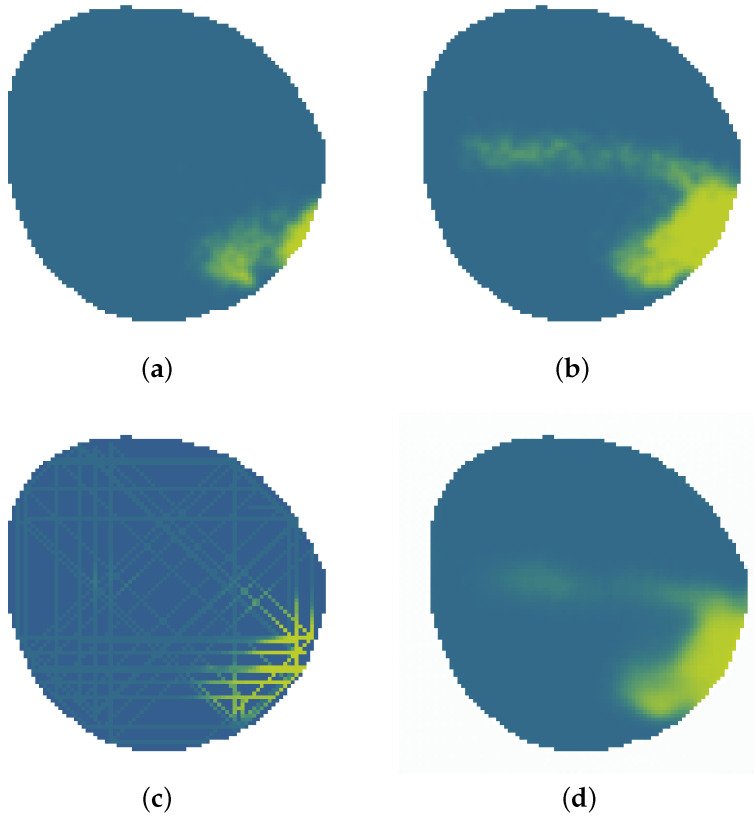
Oil spill evolution and contamination prediction problem where oil is showed in green. (**a**) Oil spill at timestamp 50. (**b**) Oil spill at timestamp 200. (**c**) Data measured by agents. (**d**) Possible estimation at timestamp 200.

The aim of the proposed variational autoencoder (VAE) is to predict and detect the evolution of an accidental water contamination, providing a visual image of the current and future states of contamination, detailing its evolution. The environment analyzed evolves dynamically, increasing in size and expanding with time. An example of an oil spill contamination accident is showed in green in Figure 1 at different timestamps.

The information available is the data gathered by a fleet of autonomous surface vehicles equipped with sensors, which are called agents from now on. This information is assumed to be incomplete and dispersed like that seen in Figure 1c. Furthermore, this information can become easily outdated as the contamination value measured by an agent is instantaneous. In previous works like [29], contamination evolution was estimated through the Gaussian process. In [26], a neural network model was used to estimate the contamination distribution at the current timestamp. An example of this estimation can be seen in Figure 1d.

In this paper, this problem is further addressed. The data gathered by the agents need to estimated not only for the current timestamp contamination distribution but also the distributions at future timestamps. The complexity added by the estimation of future states of contamination requires a contamination model. Due to the uncertainties of the water environment and the unknown factors that affect oil spill contamination, establishing a mathematical model with the available data is not possible. To solve this problem, this paper proposes a VAE as a contamination model to provide future contamination states.

The reliability of this estimation is measured using the mean squared error (MSE) of the contamination distribution. At a given coordinate, the ground-truth contamination is compared with the estimated contamination distribution, and the MSE is calculated.

### 2.2. Environment Characterization

**Figure 2:**
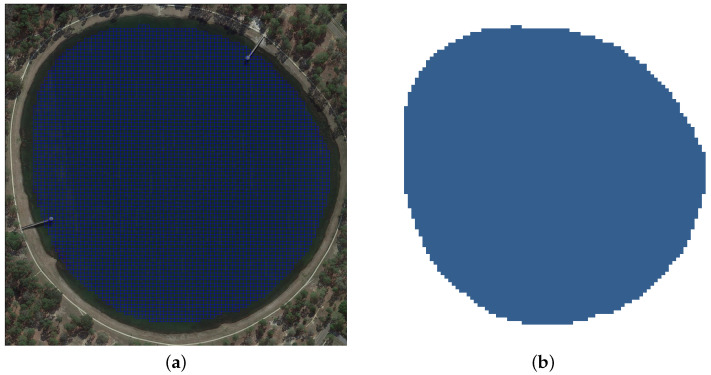
Environment characterization. (**a**) Environment grid *V* with *M* detailed. (**b**) Navigable water occupancy grid *M*.

Given a water body affected by oil spillage, the 3D environment is reduced to a 2D horizontal distribution of surface pollutants as in previous works [12], assuming that pollutants in the vertical (z-direction) are thoroughly mixed, with a negligible concentration diffusion coefficient. Space will be divided using an arbitrary-sized square grid H×W, as seen in Figure 2a. The environment is hence represented as a graph G=(V,E) defined as follows:

- $V=\{v_{i,j}|1\leq i\leq H,1\leq j\leq W\}$, where each node $v_{i,j}$ represents a specific position in the grid.
- E⊆V×V is a subset of edges that connect adjacent nodes indicating possible movements between positions. Node adjacency is defined under the assumption that the grid is 8-connected.

From the environment representation, we can analyze navigable waters. For each node $v_{i,j}$, we will assign a binary value {0, 1}, defining matrix *M* of size H×W as the occupancy of the node $v_{i,j}$ in graph *G* as seen in Figure 2b:

(1) $$M\left[i,j\right]\in \left\{0,1\right\}\;\;\;\mathrm{where}\;\;\;M\left[i,j\right]=\left\{\begin{array}{cc} 1 & \mathrm{if}\;v_{i,j}\;\mathrm{is}\;\mathrm{navigable}\\ 0 & \mathrm{otherwise} \end{array}\right.$$

### 2.3. Oil Spill Contamination Simulator

**Figure 3:**
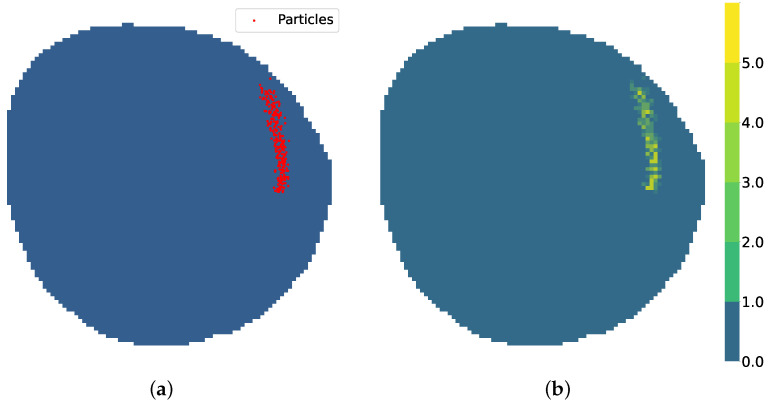
Contamination particle distribution. (**a**) Details of set of real contamination positions *B* over *M*. (**b**) Contamination particle matrix $\overset{˚}{Y}$.

Contamination is defined as the concentration of a contaminant like crude oil measured by a sensor. The aim of the simulator is to provide contamination behavior that follows the movement of real contamination. To represent the contamination in a computationally efficient way, the oil contamination in the water body is modeled as a set of discrete particles represented by a set of real positions. With *K* as the total number of contamination positions, the set *B* is defined as

(2) $$B=\{b_{k}=\left(x_{k},y_{k}\right)|k=1,2,\ldots ,K\}$$

where each element $b_{k}\in B$ represents a specific contamination position, and $\left(x_{k},y_{k}\right)$, the coordinates of $b_{k}$ in a continuous reference system. A representation of *B* over *M* can be seen in Figure 3a. A matrix $\overset{˚}{Y}$ of size H×W is defined as the contamination particle matrix:

(3) $$\overset{˚}{Y}\left[i,j\right]=\left|\left\{b_{k}\in B|\left(x_{k},y_{k}\right)\in Area\left(i,j\right)\right\}\right|$$

where each element $\overset{˚}{Y}\left[i,j\right]$ represents the amount of contamination positions contained in the $v_{i,j}$-associated area using the *B* set of contamination positions. *Area*(i,j) represents the area of node $v_{i,j}$ in *M* contained within the real coordinates that surround the node, and |·| denotes the size of the subset, the quantity of contamination positions in that area. $\overset{˚}{Y}$ is depicted in Figure 3b.

This simulator assumes that oil spill contamination must have a source from where all contamination particles originate from before moving through the water body, like in the case of water shipping [30]. With *K* as the total number of contamination sources. The set of contamination sources *S* is defined as

(4) $$S=\{s_{k}=\left(v_{ij}\right)|k=1,2,\ldots ,K\}$$

where each element $s_{k}$ represents the node where a contamination source is located. At each given time step, each contamination source liberates *Q* contamination particles at position $s_{k}$ contributing to set *B*.

In [23], a spatio-temporal model of the migration and dispersion of pollutants in a river was created using an empirical water quality hydrodynamic equation [23]:

(5) $$\frac{\partial hc}{\partial t}+u\frac{\partial hc}{\partial x}+v\frac{\partial hc}{\partial y}=\frac{\partial }{\partial x}\left(E_{x}\frac{\partial hc}{\partial x}\right)+\frac{\partial }{\partial y}\left(E_{y}\frac{\partial hc}{\partial y}\right)+H\sum S_{i}$$

where hc is the current flow, *E* is the sum of molecular dispersion coefficients, *H* is the river bottom elevation, and *S* is the sink-source term of contaminants. This equation for a mass of water can also be described mathematically as in [31]:

(6) $$b_{k}^{t+1}=b_{k}^{t}+\Delta t\left(\nu_{current}+g\cdot \nu_{wind}+\nu_{corretion}+\nu_{difusion}+\nu_{mechanical}\right)$$

This model has been further simplified to only consider the effects of the wind $\nu_{wind}$ and currents $\nu_{current}$ on the particle. The last three elements (horizontal turbulent diffusion velocity $\nu_{difusion}$, correction $\nu_{corretion}$, and mechanical spreading velocity $\nu_{mechanical}$) are represented by a random effect $\nu_{random}$ following the Brownian movement of particles. $w_{wind},w_{current}$, and $w_{random}$ are the weights associated to each effect of the movement of particles. The particle movement model is then defined as

(7) $${\hat{b}}_{k}^{t+1}=b_{k}^{t}+\Delta t\left(w_{current}\cdot \nu_{current}+w_{wind}\cdot \nu_{wind}+w_{rand}\cdot \nu_{rand}\right)$$

**Figure 4:**
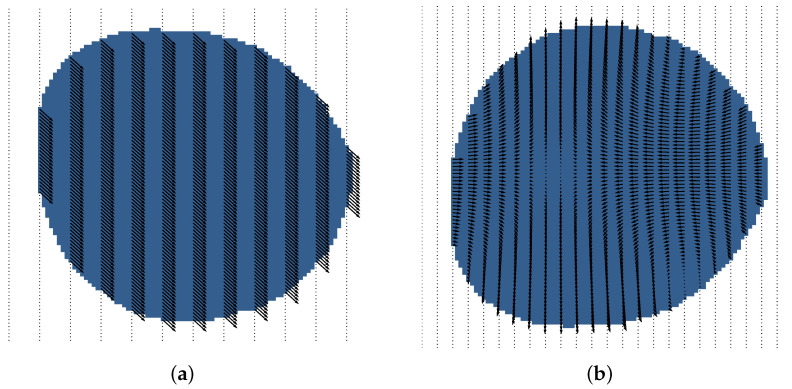
Particle movement effects. (**a**) Wind force field distribution. (**b**) Current force field distribution.

The effect of the wind is modeled as a constant 2D vector that affects the whole scenario uniformly, as seen in Figure 4a. The current effect is modeled as a constant force field that assigns each node $v_{i,j}$ a 2D vector force. This force field of currents is depicted in Figure 4b and can be modeled as

(8) $$w_{current}\left[i,j\right]=\left[\begin{array}{c} sin(i-a)*cos(j-b)\\ -cos(i-a)*sin(j-b) \end{array}\right]\;for\;\left[a,b\right]\in V$$

This simulator applies two rules before updating particle positions: No particle can be displaced beyond the water limits, staying at the last position if so. To model the concentration saturation of a node, which happens under nonlinear viscosity conditions, quantity *C* is defined as the maximum node capacity. If a contamination particle enters a node where there are already *C* particles present, the node is said to be saturated. The new particle position will update to the closest node following Algorithm 1, where *O* is defined as a set of nodes sorted in ascending order by Euclidean distance, with ${\hat{b}}_{k}^{t+1}$ as the expected new particle position for particle $b_{k}$.

(9) $$b_{k}^{t+1}=\left\{\begin{array}{cc} b_{k}^{t} & if\;{\hat{b}}_{k}^{t+1}\notin M\\ ParticleInteraction({\hat{b}}_{k}^{t+1}) & if\;{\overset{˚}{Y}}_{b_{k}^{t+1}}>=C\\ {\hat{b}}_{k}^{t+1} & otherwise \end{array}\right.$$

**Figure 5:**
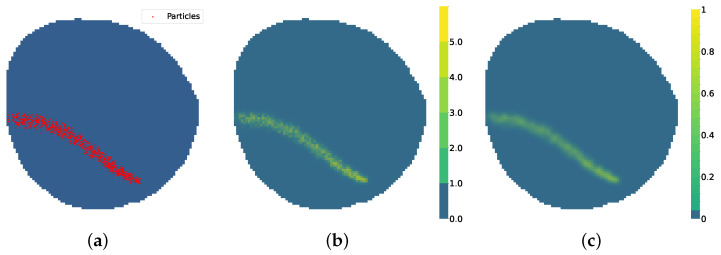
Simulator model. (**a**) Particle positions *B*. (**b**) Contamination particle matrix $\overset{˚}{Y}$. (**c**) Oil contamination concentration *Y*.

Working with discrete particles is efficient from the simulator’s point of view. However, in a real-world scenario, an oil spill cannot be measured with particles. A good approximation to a valid real-world measure is contamination concentration. Given the amount of contamination particles present in a node $\overset{˚}{Y}\left[i,j\right]$ and the maximum number of particles allowed to be present in a node *C*, node contamination density can be calculated. The shape of a real oil spill contamination area is characterized by a continuous contamination concentration [30]. The node contamination density provided by this simulator is a sparse matrix that presents sharp changes in the contamination density between adjacent nodes. To smooth the output of the simulator, a 5 × 5 kernel Gaussian mask is applied to the contamination density, providing the contamination concentration Y[i,j] as the final output, as seen in Figure 5c.
**Algorithm 1** 
ParticleInteraction
**Input:** ${\hat{b}}_{k}^{t+1}$**Output:** $b_{k}^{t+1}$**Require:** *V*   1:$\tilde{P}\leftarrow {\hat{b}}_{k}^{t+1}$   2:**for** $\overset{˚}{v_{ij}}\in O\left({\hat{b}}_{k}^{t+1}\right)$ **do**   3:    **if** $\overset{˚}{Y}\left(\overset{˚}{v_{ij}}\right)>=C$ **then**   4:        $b_{k}^{t+1}=\overset{˚}{v_{ij}}$   5:        break   6:    **end if**   7:**end for**

### 2.4. Agent Description

The vehicles that monitor the environment are defined as a fleet of *N* agents. Each agent is represented by a variable $p_{n}$, where *n* is the vehicle index.

(10) $$P=\{p_{n}|n=1,2,\ldots ,N\}$$

$p_{n}$ is characterized by several factors:

- Position ($p_{n}$): Each position of the fleet corresponds to a node. A position can be described as $p_{n}=v_{i,j}\in V$
- Speed: An agent is able to move along the 8-connected node grid. However, some agents may be able to move several nodes along the same direction.

For an agent, moving along a given node of the map, whether horizontally, vertically, or diagonally, requires one temporal unit. At any given instant, agents must stay withing navigable water nodes *M* and no more than one agent can be simultaneously situated in a node to avoid crashes between them.

Agents are able to take measures of the oil contamination concentration, be it by electrochemical or spectral sensors like multi-spectral cameras [7]. Agents that use electrochemical sensors are able to take punctual measures. This measure will be representative of the oil contamination concentration in the node. This hypothesis will hold, given that the smoothness of the contamination phenomena maintains the locality of every measurement. Multi-spectral cameras are able to cover larger areas, allowing us to measure contamination concentration in nodes adjacent to the agent.

**Figure 6:**
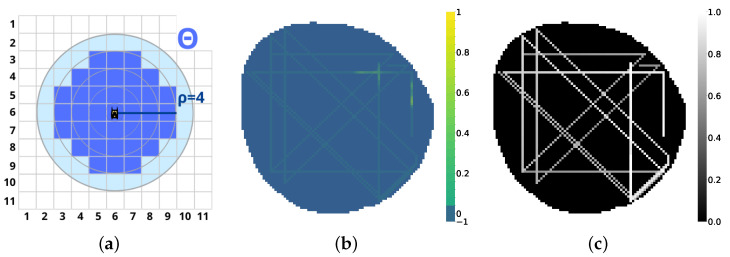
Agent Model. (**a**) Influence radius Θ. (**b**) Model contamination $\hat{Y}$ (ρ=1). (**c**) Time dependence *U* (ρ=1).

Agent monitoring sensing capability is hence characterized by an influence radius $\rho_{n}$. At any time step, the agent will provide measured data of the node occupied by it and all the nodes inside a circular area of radius $\rho_{n}$. As seen in Figure 6a, Θ is defined as the set of nodes inside the vehicle influence radius.

(11) $$\Theta \left(p_{n}\right)=\left\{v\in V|\left|v,p_{n}\right|<=\rho_{n}\right\}$$

As agents explore the map, vehicles measure the contamination concentration in the environment. $\tilde{Y}$ is defined as the measured vehicle contamination concentration model:

(12) $$\hat{Y}\left[i,j\right]\leftarrow Y\left[i,j\right]\Leftrightarrow v_{ij}\in \Theta \left(p_{n}\right)$$

This matrix is initialized to −1, as vehicles have no prior knowledge about contamination positions. If at a given instant, a node $v_{i,j}$ is contained by an agent $p_{n}$ or its influence radius $\Theta (p_{n})$, the value of $\hat{Y[i,j]}$ will be filled with instantaneous values of Y[i,j], as seen in Figure 6. As contamination positions *B* evolve dynamically, the values of $\hat{Y}$ become outdated. To provide temporal information to the vehicle contamination model $\hat{Y}$, a time dependence matrix U[i,j] is defined as

(13) $$U^{t+1}\left[i,j\right]\leftarrow U^{t}\cdot \gamma +\Theta \left(P\right)$$

This matrix is initialized to 0, denoting that the point has not been visited yet. As U[i,j] is visited by an agent or an agent is inside its influence radius $\Theta (p_{n})$, it is updated to 1, denoting that the cell has been visited recently. Then, each temporal unit U[i,j] is multiplied by a forgetting factor γ in the range (0,1). This translates to a value of U[i,j] closer to 0 the older the measure present in the contamination model $\hat{Y}\left[i,j\right]$, as seen in Figure 6c.

**Figure 7:**
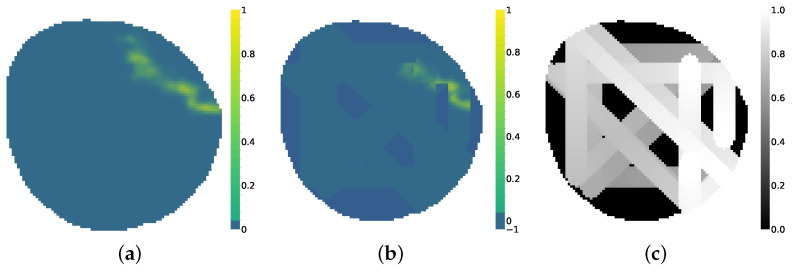
Agent exploration. (**a**) Contamination concentration *Y*. (**b**) Model contamination $\hat{Y}$ (ρ=4). (**c**) Time dependence *U* (ρ=4).

The monitoring data of a fleet of four agents with influence radius ρ=1 can be seen in Figure 6. The same fleet with influence radius ρ=4 monitoring data can be seen in Figure 7. The fine estimation of the real contamination distribution will rely upon the VAE model, as explained in the following Section. Ultimately, all environment parameters are summed up in Table 1.

### 2.5. VAE-UNet Model

**Figure 8:**
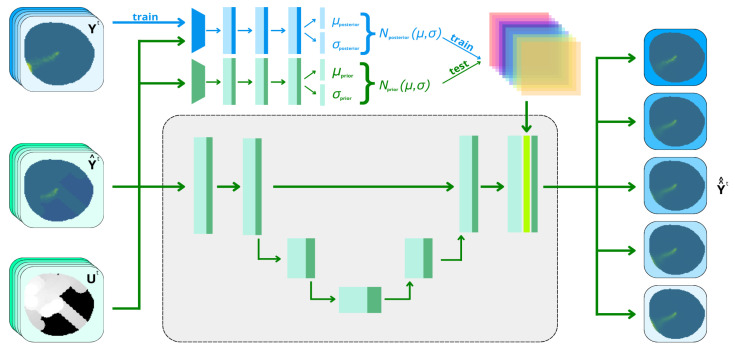
VAE-UNet architecture.

The proposed variational autoencoder architecture is an improvement of the one proposed in [26]. It is composed by a fully convolutional neural network (CNN) with an encoder and decoder phase comprising four convolutional and max-pooling layers inherited from UNet shape [27]. The variational side comprises 2 separate CNNs that produce the prior $N_{prior}\left(\mu ,\delta \right)$ and posterior $N_{posterior}\left(\tilde{\mu },\tilde{\delta }\right)$ probabilistic Gaussian distributions. The network architecture is depicted in Figure 8.

**Figure 9:**
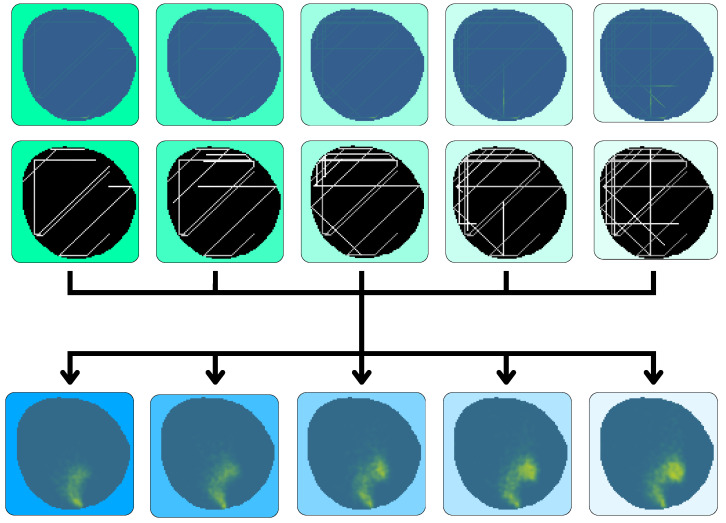
Expected inputs and outputs.

The main new developments from [26] add new channels providing past information of the contamination model in the input, aiming to obtain future states of the environment. Hence, at time *t*, the input of the VAE will contain a temporal window of the measured contamination ${\hat{Y}}^{t}$ constructed from agent samples and the time dependence $U^{t}$ associated with them. This set of 2 inputs is concatenated with *K* samples of data at present and past states $Input={\hat{Y}}^{t-k},U^{t-k},{\hat{Y}}^{t-k+1},U^{t-k+1},\ldots ,{\hat{Y}}^{t},U^{t}$k∈K. These windows can be formed by consecutive timestamps or be selected with asymmetric timestamps. The outputs of the network are the expected real contamination index at current time ${\hat{\hat{Y}}}^{t}$ and future time ${\hat{\hat{Y}}}^{t+1},\ldots {\tilde{I}}^{t+K}$. This scheme can be seen in Figure 9.

#### 2.5.1. VAE-UNet Architecture

The proposed VAE needs to learn from a vehicle model contamination matrix and extract not only the data provided but also the implicit information about the dynamics that rule the environment and invert them to provide the desired output contamination estimation. Locating an oil spillage on a map is a task that involves visual information. The use of CNNs facilitates the extraction of high-level features from the input channels and, combined with a UNet shape [27], is able to perform the image segmentation of details very well. On the other hand, a structure broadly used in neural networks when the desired output replicates the input with variations is the autoencoder [32]. This structure is able to encode the main aspects that dictate the behavior of the oil spill to a reduced dimension layer, called the latent space, and use the same rules to reconstruct the same inputs. The module in charge of reducing dimensions is called the encoder, and the one in charge of reconstructing the input is called the decoder. An enhanced version of the autoencoder, called a variational autoencoder [33], parameterizes the latent space into *n* Gaussian distributions, with its mean μ and covariance $\sigma^{2}$ being the output layers of its encoder. This approach is able to provide a structured architecture to fit the data into Gaussian distributions, with the subsequent benefits of the sampling capacity allowing us to provide as many different outputs as samples taken from these Gaussian distributions. In [28], this VAE architecture was improved through the addition of another latent state called the posterior, introducing the prior–posterior architecture.

The VAE used in this study presents 2 different latent spaces produced in the encoder–decoder phase. The one generated by the prior has as inputs the measured contamination $\hat{Y}$ and time dependence *U*, providing a latent space $N_{prior}$ originated from the minimization of the prediction error from the partial observations. The second latent space $N_{posterior}$ has as inputs not only the partial observations but also the actual real contamination concentration *Y*. The information provided by training with the desired output makes the network learn from data not available originally, providing a lower loss and hence, a better latent space. However, complete contamination information is not available in a real-world experiment, with the prior network being the only feasible solution. The aim of this architecture is to track both latent spaces during training and try to reduce the Kullback–Leibler divergence [28] present between the latent spaces of the prior and posterior network. In this way, the prior is able to induce the data only provided to the posterior. This makes the generated estimations provided by the VAE follow behaviors closer to the ground-truth when partial observations are provided. A scheme of the architecture is shown in Figure 8.

#### 2.5.2. VAE-UNet Loss Function

The loss function here is an adaptation of the loss proposed in [34]. The output of the VAE ($\hat{\hat{Y}}$) must match the real ground truth (*Y*). However, during training, neural networks are biased to first minimize the largest loss [35]. Due to the nature of a temporal prediction, the farther it is into the future, the higher the probability to miss in the prediction. This motivates the network to minimize loss in most future predictions (higher by origin) and less in the near future. To avoid this and improve the accuracy of close future predictions, the MSE of the future predictions is weighted with a forgetting factor ϕ∈(0,1]. The reconstruction loss at instant t+k is thus defined as

(14) $$L_{recon}=\sum \mathrm{MSE}\left(Y^{t+k},{\hat{\hat{Y}}}^{t+k}\right)\cdot \phi^{k}\;\;k\in N$$

As mentioned before, the latent space is doubled by 2 variational latent spaces. The output of the network is composed using the posterior latent space during training (blue lines in Figure 8) and prior latent space during testing (green lines in Figure 8). The divergences between both latent spaces leads to the definition of the Kullback–Leibler (KL) divergence loss.

(15) $$L_{KL}=KL\left(N_{prior}\left(\mu ,\delta \right),N_{posterior}\left(\mu ,\delta \right)\right)$$

**Figure 10:**
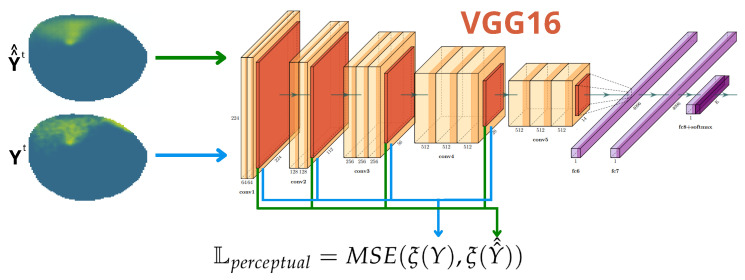
Depiction of the feature comparison performed in $L_{perceptual}$.

Lastly, As the VAE needs to process an image as input and provide an image as output, a style transfer strategy [36] is adopted utilizing the VGG16 [37]. The VGG16 is a fully trained CNN for image recognition on a huge dataset. Through studying this network, it is observed that the initial layers contain the low-level features of the image (color, edges, texture, etc.), and deeper layers contain higher-level features (objects and their arrangement in the input image) [36]. In [36], it was demonstrated that pixel-by-pixel comparisons, like the one we performed with $L_{recon}$, show a low performance when training a new neural network using a small dataset. However, if training is assisted using a fully trained network like the VGG16, it results in a much better-performing network. This is defined as style transfer [36] and is a strategy used in image transformation problems. The VGG16 is used to compare the output of the proposed VAE-UNet against the actual ground truth using the features extracted by the VGG16. By doing this, we encourage the output oil spill to cover the same nodes that are polluted in the ground truth, preserving the same spatial structure. The mean square error (MSE) of the VGG16 layers relu1_2,relu2_2,relu3_3, and relu4_3 is defined as Perceptual Loss, as described in Figure 10.

(16) $$L_{perceptual}=\mathrm{MSE}\left(\xi \left(Y\right),\xi \left(\hat{\hat{Y}}\right)\right)$$

The total loss of the network is defined as the weighted sum of all 3 losses.

(17) $$L=\omega_{recon}L_{recon}+\omega_{KL}L_{KL}+\omega_{perceptual}L_{perceptual}$$

### 2.6. Agent Planner

The fleet model plays a crucial role in the modeling of the contamination. The input of the VAE is directly related to the fleet’s information-gathering performance. It is necessary to address the model at the same time that the path planner is designed. The unavoidable consequence of an ill-designed model is poor monitoring and decision making. From the model perspective, poor information acquisition will result in a catastrophic scarcity of examples to learn from.

Agent information policies have been broadly studied [13], and the optimal policy is out of the scope of this paper. The aim of this study was to use the VAE to predict future states of contamination from partial observations that are assumed to provide relevant enough data of the contamination source. The proposed policy was selected to be a safe random coverage policy that provides enough information without considering the optimal policy in the long range. This puts the focus on the necessary objective of obtaining a model that works even when the policy is not perfect. Consequently, the model will be robust enough to serve other purposes in other scenarios. At the start, agents choose a random direction in the 8-connected grid to move toward. The selection takes into account that the agent will stay within navigable water *M* limits and the destination cell is not already occupied by another agent. When an agent movement will cause the agent to leave *M* or crash with another agent, the agent chooses a new safe random direction in the 8-connected grid. To avoid the overlapping of positions and possibilities of agents occupying the same node due to simultaneous movement, agents follow a priority order defined randomly at the start of the simulation, where an agent executes the policy just after the previous one has established a target position.

## 3. Results

**Figure 11:**
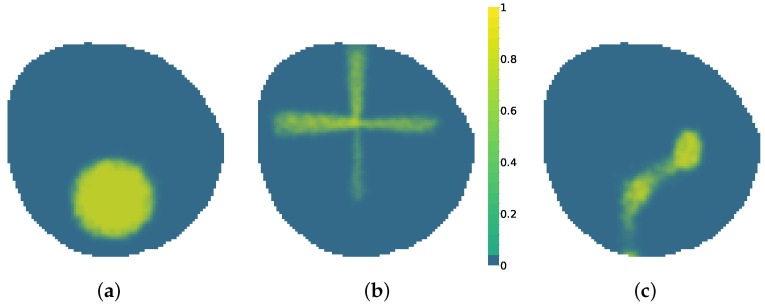
Oil spill behaviors. (**a**) Circular expansion. (**b**) Triangular diffusion. (**c**) Linear dispersion.

Experiments were performed using a 97 × 93 node scenario in a circular shape. The simulator was used offline to create datasets for $\hat{Y^{t}}$ and $U^{t}$ as inputs of the network and $Y^{t}$ as the ground truth of the scenario and the value we want to compare to as output from the variational autoencoder (VAE). To evaluate the performance of the VAE in different environments, the simulator was configured to create scenarios where oil spill evolution can present three different behaviors: Linear dispersion, currents and wind affect particles moving in a general direction, allows erratic behaviors, as seen in Figure 11c. Circular expansion, wind and currents forces are minimal and the sources have a high flow of particles, allowing for particles to grow in a circular shape as seen in Figure 11a. Triangular diffusion is a rule-based behavior that mimics an oil spill caused by a flow coming from a broken cross pipe that follows a cross shape, as seen in Figure 11b. Oil presents a fast release that slows down once it has been liberated to the water body. This last environment presents the most artificial behavior but adds more complexity to the problem. The simulator (https://github.com/AloePacci/cpp_oil_simulator, accessed on 11 January 2025) and VAE (https://github.com/AloePacci/VAEPOCTEWE, accessed on 11 January 2025) codes are available on GitHub.

**Figure 12:**
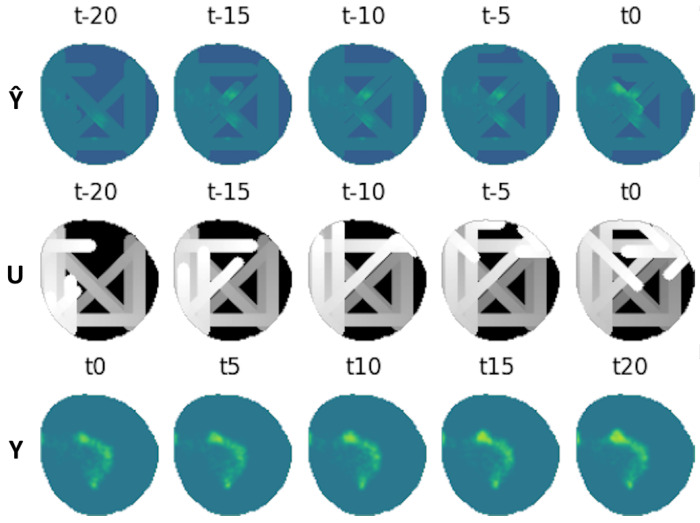
Dataset example containing inputs $\left({\hat{Y}}^{t},U^{t}\right)$ for ρ=4 and ground truth $\left(Y^{t}\right)$.

The VAE was configured to have five window frames $\left({\hat{Y}}^{t},U^{t}\right)$ as inputs expatiated uniformly five time steps between each other ranging from $t_{-20}$ to $t_{0}$, and another five frames as output ranging from $t_{0}$ to $t_{20}$. Several datasets were created for each of the oil spill behaviors; 20,000 different contamination scenarios were synthesized for training, 4000 for testing and 200 for validation. These include monitoring situations with agents equipped with electrochemical sensors, with influence radius ρ=1, and agents equipped with cameras, with influence radius ρ=4. An example of a contamination instance dataset can be seen in Figure 12.

All simulations and training were carried out on a server running Ubuntu 22.04.4 LTS (Universidad de Sevilla, Sevilla, Spain), equipped with an Intel Dual Xeon Gold 5220R CPU 2.20 GHz, 192 GB of RAM and two GPUs: Nvidia Quadro A4000 48 GB and Nvidia RTX 3090 25 GB. Training loss was calculated using Equation (17) and forgetting factor ϕ=0.9. The hyperparameters $\omega_{recon},\omega_{KL},\omega_{perceptual}$ and the learning rate lr were optimized utilizing Optuna [38] to minimize the reconstruction loss $L_{recon}$ in order to address the final objective of model accuracy.

Given the datasets and different combinations of agents and oil spill evolution behaviors, different networks were trained to calculate the cross losses and validate the effectiveness and generalizing capabilities of the proposed VAE. Thus, results were divided into a fleet of agents characterized by an influence radius ρ=1 and another characterized by an influence radius ρ=4. For each fleet of agents, four different models were trained: one for each of the oil spill behaviors for cross validation, and one containing a fusion of all three oil spill behaviors, from now on called the generalized network. The weights were chosen for the network at the epoch that showed the lowest test loss.

### 3.1. Performance Metrics

As mentioned previously, the aim of the VAE-UNet is to provide future states of oil spill contamination. The baseline taken for comparison to evaluate the performance of the network is the static approach, where the environment is considered non-dynamic and the expected future state of the contamination position is equal to the current one ${\hat{\hat{Y}}}^{t+k}={\hat{Y}}^{0}$k∈N. The loss at time step 0 at areas recently visited by agents is minimal. However, in areas with data measured several steps ago, or with predictions at future timestamps, the error using this approach increases at a high rate. The visual of this loss establishes this baseline as a solution that underperforms but provides a valid estimation.

**Figure 13:**
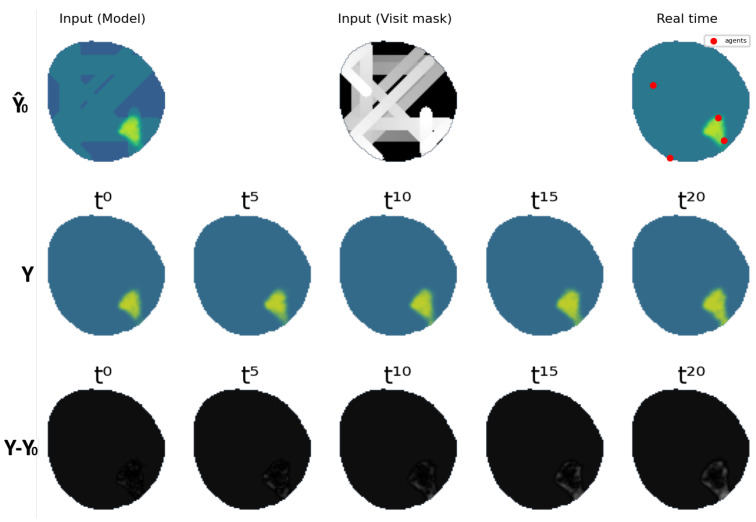
Baseline error $\left(Y^{t}-Y^{0}\right)$.

Figure 13 shows the results of evaluating the reconstruction loss MSE $\left(Y^{t}-Y^{0}\right)$ before using the baseline approach. The loss incurred by the VAE-UNet was calculated with respect to the baseline and expressed in a percentage value of the baseline loss.

### 3.2. Fleet with ρ=1

**Figure 14:**
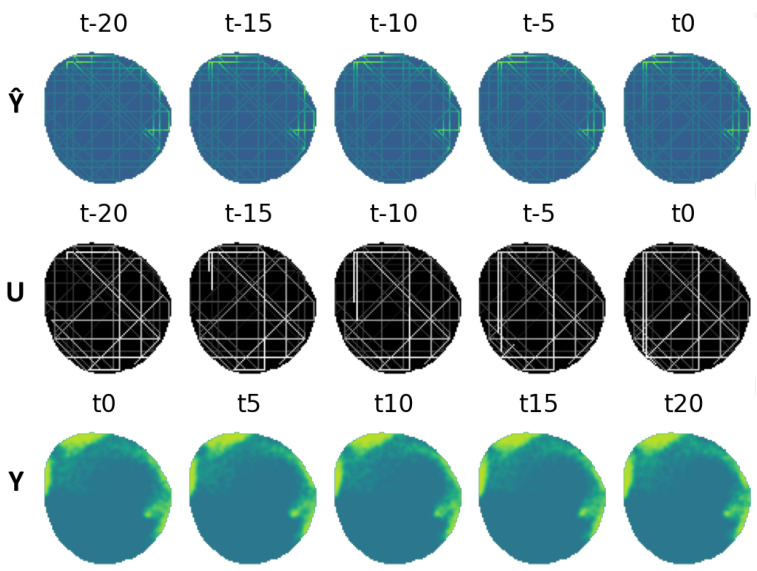
Dataset example containing inputs $\left({\hat{Y}}^{t},U^{t}\right)$ for ρ=1 and ground truth $\left(Y^{t}\right)$.

This fleet is characterized by an influence radius ρ=1 and is able to take measurements in the nodes where the agents are currently located at, like the one used in [39] equipped with electrochemical sensors. It is made up of four different agents that are able to move through three cells each time step. An example of the dataset can be seen in Figure 14.

**Figure 15:**
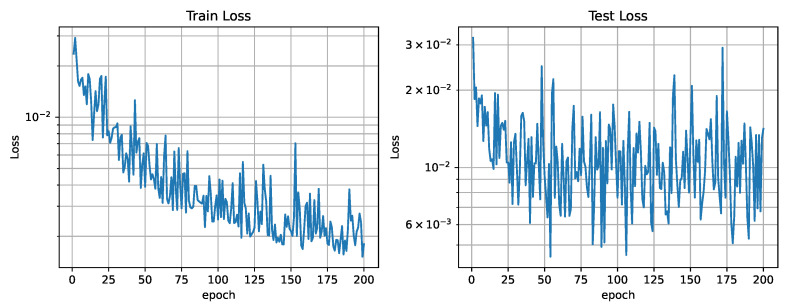
Training and test loss curves (ρ=1).

**Figure 16:**
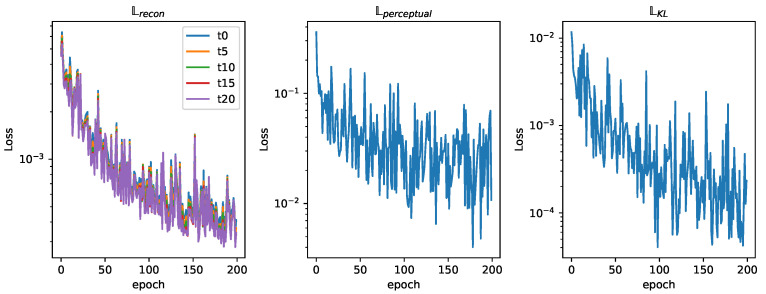
Training loss curves (ρ=1).

The networks training loss curve shows a high descending slope that stabilizes around epoch 100, as seen in Figure 15. A value of 200 epochs for training was considered sufficient. Figure 16 shows the relation between the three different losses during training. In analyzing the reconstruction loss $L_{recon}$ curve, the loss associated with each of the future estimations shows a similar value despite the loss reduction applied to future predictions. This justifies the assumption taken before that the further the network looks into the future, the higher the loss.

In Table 2, the reconstruction loss value of each trained network is shown. The network trained using only triangular diffusion data shows the lowest loss. This could be due to the simplicity of the contamination behavior for this case. It is followed by circular expansion; being simpler, it presents no effects of the wind or currents. Lastly, the linear dispersion case shows the highest loss. This could be due to the high variety and random evolution of this contamination behaviour. The generalized network, presenting data from all the different datasets, presents a middle value.

Once the different networks were trained, their performances were evaluated. Table 3 shows the reconstruction loss results of evaluating each trained network against each of the different validation datasets. In view of the results, the solution presented in this paper is able to provide a prediction of the evolution of an oil spill with an error of less than 10% of the naive baseline approach for each assigned contamination behaviour. This shows that the network is able to predict oil spill evolution with high accuracy in environments similar to those it was trained with.

To test the adaptability of the trained VAE-UNet in unseen behaviour, the network was evaluated against datasets in which the oil spill behaves very differently compared to the dataset that it was trained on. The results show that the VAE behaves better than the baseline prediction in all individual cases, except for the circular expansion cross-loss against the triangular diffusion case, which underperformed. This occurs mostly due to overfitting, as there is no wind or current effect in the circular expansion dataset. The opposite can be seen in the linear dispersion network, where contamination particles evolve in diverse ways, allowing for a better adaptability and lower cross-losses. A special case is triangular diffusion error, where the generalized VAE performs better than in triangular case in its own error. This could be due to loss hyperparameters being optimized for the generalized case and a better understanding of particle behavior due to a more varied dataset.

Lastly, Table 4 shows the evolution of $L_{recon}$ along the different prediction timestamps for each VAE-UNet trained through the complete validation dataset. The values show that as the VAE-UNet predicts further into the future, the higher the error in the prediction. The increase in this loss is higher in the specific contamination behaviors of VAE-UNets due to them not being trained for the whole dataset. However, the loss value remains low, presenting a better estimation than the baseline. The Supplementary Material includes a video showing the evolution of the VAE-UNet as the fleet explores the map.

### 3.3. Fleet with ρ=4

**Figure 17:**
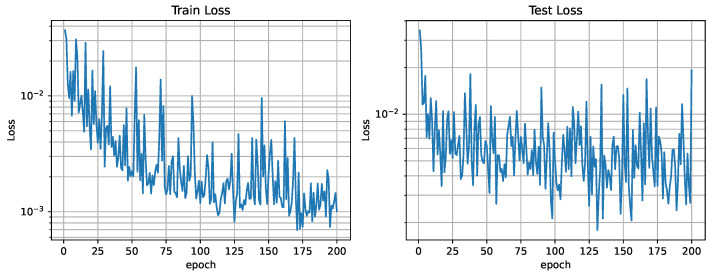
Training and test loss curves (ρ=4).

**Figure 18:**
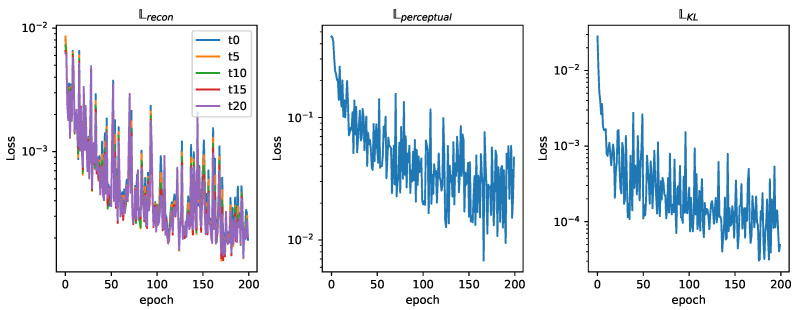
Training loss curves (ρ=4).

The fleet is characterized by an influence radius ρ=4; this could be the case of agents equipped with spectral sensors like the ones present in [40]. It is made up of four different agents that are able to move through three cells each time step. An example of this fleet’s dataset can be seen in Figure 12. This fleet is able to provide contamination information about nodes adjacent to the agents in a four-node radius, providing more information than the one equipped with electrochemical sensors, resulting in a lower loss, as seen in Figure 17. The analysis of the reconstruction loss for each time step in Figure 18 shows similar values despite the loss reduction. This result enhances the assumption that the further into the future the estimation, the higher the error committed by the VAE in the prediction. Table 5 presents the training and testing reconstruction losses. The losses present the same relationships. However, due to having more information, the magnitude of the loss is lower.

The performance of the network was evaluated, and the results are shown in Table 6. The static approach selected as the baseline provided by this fleet has more information, providing an estimation loss that is six times lower on average. The VAE is able to process the new information to provide better estimations. However, even though the absolute loss values were reduced. The cross-losses show a performance worse than those of the baseline in environments different from those in the trained cases. This overfit is more present in the circular expansion case trained without the influences of the wind or currents. The opposite is seen in the linear dispersion case, presenting a more varied training dataset and environment effects.

Table 7 shows the evolution of $L_{recon}$ along the different prediction timestamps through the complete validation dataset. The overfit can be easily seen in the circular expansion network. The rest of the trained networks present loss values lower than those of the baseline. The generalized dataset presents the lowest reconstruction loss value, being the network trained with the most varied dataset. These results proclaim that the more varied the dataset, the better and the more robust the network, leading to better adaptation to unknown environments and lower losses.

**Figure 19:**
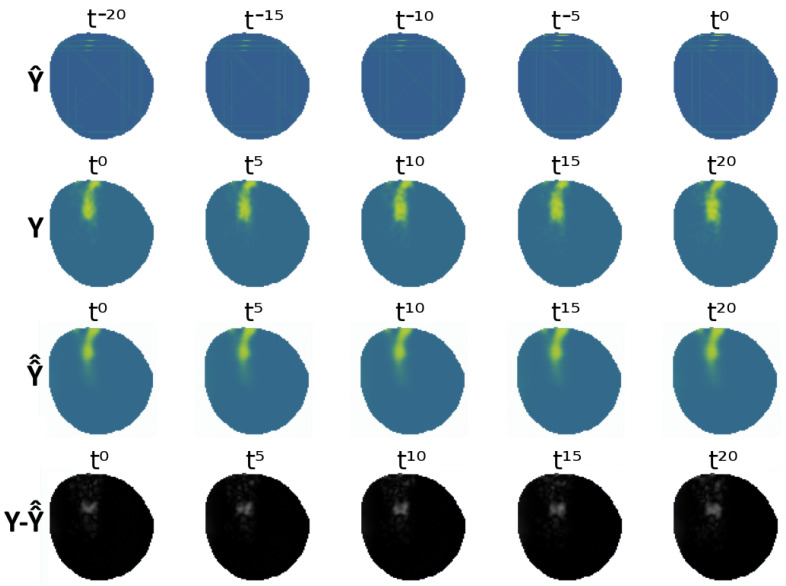
Comparison of VAE (generalized) output against real ground truth.

**Figure 20:**
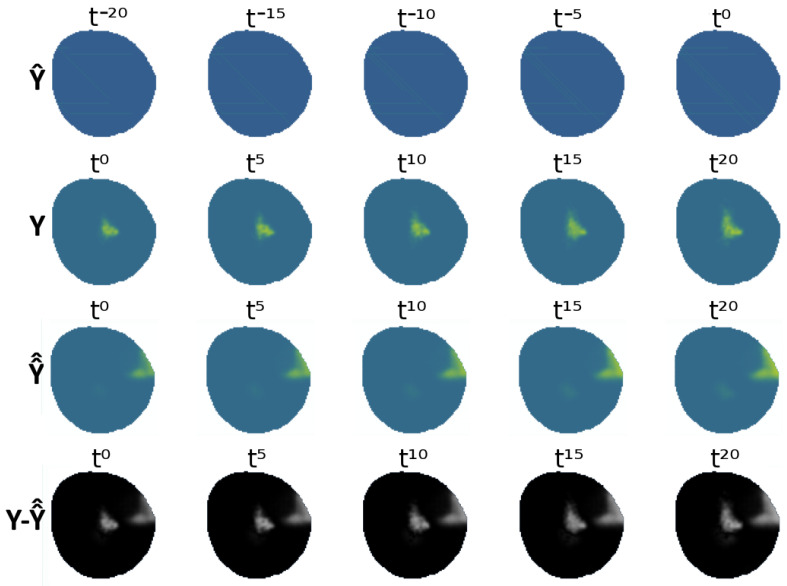
Comparison of VAE output against real ground truth, unfavorable case.

A visual representation of the VAE-UNet generalized network for the fleet equipped with electrochemical sensors can be seen in Figure 19, showing the partial input $\hat{Y}$ and the output of the VAE $\hat{\hat{Y}}$ against the ground-truth data *Y* and the difference between both $\hat{\hat{Y}}-Y$. As mentioned previously, the agent policies are not the objective of this study. Figure 20 shows an example where agents have yet to discover the oil spill contamination. The VAE-UNet predicts contamination to be in an erroneous position. This addresses the effect of the fleet’s information-gathering performance present both during testing and training in the modeling of the contamination.

**Figure 21:**
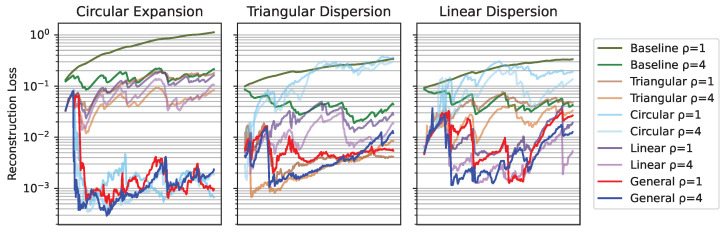
Reconstruction loss during a synthesized oil spill contamination accident.

Figure 21 shows the evolution of the different losses along three different oil spill environments. Initially, reconstruction loss increases until the contamination area is detected and then decreases sharply. The generalized network presents instances where the fleet with ρ=1 presents a lower loss than ρ=4. This is due to the planner policy of the agents that provides different monitoring information to each fleet.

## 4. Conclusions

This paper proposes a variational autoencoder to predict the evolution of oil spill contamination in water bodies from partial observations. To assess performance, it was tested on several scenarios presenting three different simulated oil contamination environments: circular expansion, presenting minimal wind and current forces; triangular diffusion, where contamination is exposed to biased currents; and linear dispersion, allowing random behaviors with high wind and current effects. Furthermore, the test was duplicated using two fleets of autonomous surface vehicles with different monitoring capabilities: a fleet equipped with electrochemical sensors able to take punctual measurements and a fleet equipped with spectral cameras able to monitor an area close to the vehicle.

According to Table 4 and Table 7, the validation results of the proposed generalized VAE show a prediction loss as low as 3.51%, the baseline at current time, by the fleet equipped with electrochemical sensors, and as high as 8.21%, the baseline 20 time steps into the future, by the fleet equipped with spectral cameras. The magnitude of this loss increases with the age of the predictions, presenting an increase the further into the future that the predictions are made. The overfit of the network to the data trained on was tested using networks trained with datasets presenting only one of the three available environments. The results show a lower loss at the specific environments and a higher loss at different ones. A further study showed that this overfit decreases when the network is trained with a more varied dataset, presenting validation losses as high as six times the baseline for the circular expansion case in the fleet equipped with spectral cameras, or 11.73% for the baseline, in the linear dispersion case with the fleet equipped with electrochemical sensors. Thus, it is expected that the proposed generalized network trained with a varied dataset performs very well in new environments.

The gathering performance of the agents affects the proposed VAE in two different ways. The fleet equipped with spectral cameras is able to cover a wider monitoring area, providing more monitoring data and allowing for a reconstruction loss six times lower on average. Furthermore, the wider coverage allows for detecting the contamination position with more certainty. The path-planning policy is random, presenting cases where the vehicles have not detected any contamination and the prediction erroneously locates the contamination. Thus, in a monitoring scenario, the initial losses of the proposed VAE show an underperforming solution.

Future lines of work diverge into two lines of investigation. On the one hand, an analysis of the effect of the proposed VAE-UNet structure on informative path planning should be performed, providing numeric data of the effects of taking a future state of contamination particles into account in agent policy estimation. On the other hand, the limits of the VAE should be addressed, evaluating the effects on the prediction accuracy of different agent policies and the input requirements regarding number and age of window frames.

## Figures and Tables

**Table 1:** Environment description.

Parameter	Description
G(V,E)	Environment graph.
$V,v_{ij}$	Set of environment nodes.
*E*	Set of paths between nodes.
M[i,j]	Navigation matrix. Indicates if a node i,j can be visited.
$\delta_{t}$	Internal simulator time constant.
$B,b_{k}$	Set of contamination particles. Each particle has a position and is associated with a node *v*.
$S,s_{k}$	Set of source points. Each source point has a position and is associated with a node *v*.
*Q*	Pollutant liberated from the scenario by the contamination source each time step.
$\overset{˚}{Y}\left[i,j\right]$	Contamination particle matrix.
*C*	Maximum number of particles per node.
$Y\left[i,j\right],\hat{Y}\left[i,j\right]$	Contamination concentration matrix and vehicle model of the contamination particle matrix.
$w_{wind},\nu_{wind}$	Wind gain and wind force vector.
$w_{currents},\nu_{currents}$	Current gain and current force field.
$w_{random},\nu_{random}$	Brownian movement gain and Brownian movement force effect.
$P,p_{j}$	Fleet of agents. Set of nodes where a vehicle is present.
ρ	Maximum distance of water measurement.
*N*	Number of agents present in the fleet.
$\Theta (p_{j})$	Set of nodes inside a distance ρ.
*O*	Set of nodes sorted in ascending order by Euclidean distance.
U[i,j]	Matrix that indicates the age of the data associated with the value of node $\hat{Y}\left[i,j\right]$.

**Table 2:** Train and test reconstruction loss (fleet ρ=1).

VAE-UNet	Test Loss	Train Loss
Circular expansion	$5.216\times 10^{-3}$	$1.449\times 10^{-3}$
Triangular diffusions	$3.479\times 10^{-3}$	$1.624\times 10^{-3}$
Linear dispersion	$12.394\times 10^{-3}$	$10.139\times 10^{-3}$
Generalized	$6.637\times 10^{-3}$	$2.124\times 10^{-3}$

**Table 3:** Cross-losses VAE-UNet architecture (fleet ρ=1).

VAE-UNet	Circular Expansion Environment Loss	Triangular Diffusion Environment Loss	Linear Dispersion Environment Loss
Circular Expansion	1.54%	100.16%	74.22%
Triangular Diffusions	20.34%	4.71%	20.76%
Linear Dispersion	17.48%	11.73%	7.71%
Generalized	1.82%	4.53%	8.72%
Baseline	0.8551	0.3194	0.2815

Percentages were calculated with respect to the baseline.

**Table 4:** VAE-UNet performance comparison by time step (fleet ρ=1).

VAE-UNet	Loss $t_{0}$	Loss $t_{5}$	Loss $t_{10}$	Loss $t_{15}$	Loss $t_{20}$
Circular Expansion	36.04%	37.66%	39.08%	40.59%	42.27%
Triangular Diffusions	14.65%	15.7%	16.73%	17.66%	18.45%
Linear Dispersion	12.41%	13.25%	14.11%	14.96%	15.82%
VAE-UNet Generalized	3.51%	3.68%	3.84%	4%	4.16%
Baseline	1.5173	1.5687	1.6205	1.6726	1.7252

Percentages were calculated with respect to the baseline.

**Table 5:** Train and rest reconstruction loss (fleet ρ=4).

VAE-UNet	Test Loss	Train Loss
Circular Expansion	$0.930\times 10^{-3}$	$0.756\times 10^{-3}$
Triangular Diffusions	$1.719\times 10^{-3}$	$1.427\times 10^{-3}$
Linear Dispersion	$7.580\times 10^{-3}$	$1.057\times 10^{-3}$
Generalized	$3.707\times 10^{-3}$	$1.249\times 10^{-3}$

**Table 6:** Cross-losses of VAE-UNet architecture (fleet ρ=4).

VAE-UNet	Circular Expansion Environment Loss	Triangular Diffusion Environment Loss	Linear Dispersion Environment Loss
Circular Expansion	4.31%	386.17%	601.95%
Triangular Diffusions	43.26%	18.09%	146.96%
Linear Dispersion	46.38%	53.41%	47.86%
Generalized	4.11%	16.77%	60.90%
Baseline	0.1615	0.0479	0.0233

Percentages were calculated with respect to the baseline.

**Table 7:** VAE-UNet Performance comparison by time step (fleet ρ=4).

VAE-UNet	Loss $t_{0}$	Loss $t_{5}$	Loss $t_{10}$	Loss $t_{15}$	Loss $t_{20}$
Circular Expansion	78.00%	85.32%	91.31%	96.34%	100.96%
Triangular Diffusions	27.17%	28.36%	29.64%	31.18%	32.56%
Linear Dispersion	25.34%	27.58%	29.75%	31.81%	33.76%
VAE-UNet Generalized	7.19%	7.49%	7.75%	7.99%	8.21%
Baseline	0.2470	0.2701	0.2948	0.3206	0.3474

Percentages were calculated with respect to the baseline.

## Data Availability

Data are contained within the article and Supplementary Material. The dataset can be downloaded from https://bender.us.es/acasado/dataset-vae (accessed on 14 February 2025).

## Supplementary Materials

A video was added as Supplementary Data to provide a better visual of the VAE-UNet performance: https://youtu.be/xtE6pfyCSVo (accessed on 14 February 2025). Video S1: Variational autoencoder tests fleet electrochemical sensors. https://youtu.be/KH9hu6ksXp8 (accessed on 14 February 2025). Video S2: Variational autoencoder tests fleet spectral sensors.

## Abbreviations

The following abbreviations are used in this manuscript:

2D	Two Dimensional
AIPP	Adaptative Informative Path Planning
ASV	Autonomous Surface Vehicle
MDPI	Multidisciplinary Digital Publishing Institute
DOAJ	Directory of open access journals
TLA	Three letter acronym
LD	Linear dichroism
VAE	Variational Autoencoder
CNN	Convolutional Neural Network
SDG	Sustainable Development Goal
MSE	Mean Square Error
KL	Kullback-Leibler
